# Responses of sympatric canids to human development revealed through citizen science

**DOI:** 10.1002/ece3.6567

**Published:** 2020-07-15

**Authors:** Kenneth F. Kellner, Jacob E. Hill, Mariela G. Gantchoff, David W. Kramer, Amanda M. Bailey, Jerrold L. Belant

**Affiliations:** ^1^ Camp Fire Program in Wildlife Conservation State University of New York College of Environmental Science and Forestry Syracuse New York USA; ^2^ New York State Department of Environmental Conservation Albany New York USA

**Keywords:** *Canis latrans*, Citizen science, coyote, hunters, land use, red fox, *Vulpes vulpes*

## Abstract

Measuring wildlife responses to anthropogenic activities often requires long‐term, large‐scale datasets that are difficult to collect. This is particularly true for rare or cryptic species, which includes many mammalian carnivores. Citizen science, in which members of the public participate in scientific work, can facilitate collection of large datasets while increasing public awareness of wildlife research and conservation. Hunters provide unique benefits for citizen science given their knowledge and interest in outdoor activities. We examined how anthropogenic changes to land cover impacted relative abundance of two sympatric canids, coyote (*Canis latrans*), and red fox (*Vulpes vulpes*) at a large spatial scale. In order to assess how land cover affected canids at this scale, we used citizen science data from bow hunter sighting logs collected throughout New York State, USA, during 2004–2017. We found that the two species had contrasting responses to development, with red foxes positively correlated and coyotes negatively correlated with the percentage of low‐density development. Red foxes also responded positively to agriculture, but less so when agricultural habitat was fragmented. Agriculture provides food and denning resources for red foxes, whereas coyotes may select forested areas for denning. Though coyotes and red foxes compete in areas of sympatry, we did not find a relationship between species abundance, likely a consequence of the coarse spatial resolution used. Red foxes may be able to coexist with coyotes by altering their diets and habitat use, or by maintaining territories in small areas between coyote territories. Our study shows the value of citizen science, and particularly hunters, in collection of long‐term data across large areas (i.e., the entire state of New York) that otherwise would unlikely be obtained.

## Graphical abstract

We examined how anthropogenic changes to land cover impacted relative abundance of two sympatric canids, coyote (*Canis latrans*), and red fox (*Vulpes vulpes*) at a large spatial scale. We found that the two species had contrasting responses to development, with red foxes positively correlated and coyotes negatively correlated with the percentage of low‐density development. Red foxes also responded positively to agriculture, but less so when agricultural habitat was fragmented.

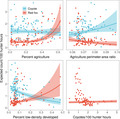

## 1 INTRODUCTION

With a growing human population, it is increasingly important to determine how anthropogenic activities can influence wildlife populations and communities (McKinley et al., 2017; Nichols & Williams, 2006). However, detecting anthropogenic impacts is difficult in many systems due to a lack of appropriate data (Magurran et al., 2010). Long‐term, large‐scale data are particularly useful because it allows for the documentation of baselines and detection of broad‐scale changes in ecological patterns that may be missed using sparse or small‐scale data sets (Lindenmayer & Likens, 2010). Despite the obvious benefits, long‐term studies remain relatively uncommon due to the costs and logistics required, especially for wide‐ranging and cryptic species (Parsons, Goforth, Costello, & Kays, 2018). One solution to overcome this problem is through citizen science, in which members of the public participate in scientific work (Bonney et al., 2009). Citizen science provides a means to achieve substantial data collection and data processing needs while also contributing to the awareness, knowledge, and understanding of the non‐science public about the practice of science and the relevance of scientific outcomes (Parrish et al., 2019). Particularly in wildlife biology, it can increase the spatial or temporal scale of research projects while increasing the public's interest and knowledge toward wildlife and the environment (Frigerio et al., 2018).

Despite issues such as incomplete reporting and skewed sampling (Tye, McCleery, Fletcher, Greene, & Butryn, 2017), citizen science data can yield robust inferences when analyzed rigorously (e.g., Davies, Stevens, Meekan, Struve, & Rowcliffe, 2012; Kéry, Gardner, & Monnerat, 2010). Standardized monitoring schemes result in the highest data quality; however, opportunistic data collection is the next best option for long‐term studies, overcoming uneven sampling with large amounts of data (Isaac, van Strien, August, de Zeeuw, & Roy, 2014). Studies based on field work are particularly challenging, due to potentially risky field conditions for researchers or the need for frequent sampling (McKinley et al., 2017). However, some subsets of the public (e.g., birders, hunters, hikers) are well‐suited for this type of data collection because they regularly visit outdoor areas and have both the physical skills and the interest to participate in outdoor activities. Hunter indices, for example, have been used for decades to monitor wildlife population trends in the United States (e.g., Haskell, 2011) and Europe (e.g., Ericsson & Wallin, 1999).

North America has the highest abundance of hunters of any region in the world (Sharp & Wollscheid, 2009), providing a unique opportunity to use hunter‐collected data to investigate broad ecological questions. The widespread occurrence of hunting facilitates use of such data to study species such as carnivores, which are generally difficult to detect given their low densities and secretive nature (Rich et al., 2013). While the geographic ranges of many North American carnivores have contracted over the past century, ranges of red foxes (*Vulpes vulpes*) and coyotes (*Canis latrans*) have increased from their historic geographic ranges by 16% and 40%, respectively (Laliberte & Ripple, 2004). Both species have a high degree of habitat and diet flexibility, which enables them to occupy habitats across a spectrum of human influences, ranging from undeveloped to highly urbanized (Bateman & Fleming, 2012).

Human alterations of the landscape are a key factor promoting range expansions of coyote and red fox (Gompper, 2002; Statham, Sacks, Aubry, Perrine, & Wisely, 2012). Coyotes frequently select early successional habitat, such as old fields, due to cover availability and high abundance of food including small mammals and plants (Holzman, Conroy, & Pickering, 1992; Richer, Crête, Ouellet, Rivest, & Huot, 2002; Schrecengost, Kilgo, Ray, & Miller, 2009). In New York State, coyote expansion is attributed in part to an increase in abandoned farmlands (Fener, Ginsberg, Sanderson, & Gompper, 2005). Active fields may also provide food and cover depending on the crop (Andelt & Andelt, 1981; Hinton, van Manen, & Chamberlain, 2015). Conversion of land to agriculture may further benefit coyotes due to increased prevalence of edge habitat, which can provide prey and facilitate travel (Theberge & Wedeles, 1989). Rural areas may thus support higher numbers of coyotes compared to forested landscapes (Richer et al., 2002). Coyotes also inhabit more developed areas where they can find a variety of anthropogenic food (Murray et al., 2015), but may be deterred from some such habitats due to conflict potential with humans (Gosselink, Van Deelen, Warner, & Joselyn, 2003).

Dietary overlap between coyotes and foxes can result in similarities regarding habitat selection (Theberge & Wedeles, 1989). Like coyotes, red foxes may use urban and agricultural habitats (e.g., Gosselink et al., 2003; Harris & Smith, 1987). However, red fox habitat selection is also sometimes influenced by coyote predation risk in areas where they are sympatric (Holt & Polis, 1997; Sargeant & Allen, 1989). Increases in coyote abundance can depress red fox populations, either through competition or direct killing (Levi & Wilmers, 2012). Coyotes may also displace red foxes from preferred habitats, leading them to select habitats avoided by coyotes, such as areas of higher human density (Gosselink et al., 2003; Randa & Yunger, 2006).

Habitat selection occurs at multiple spatial scales, ranging from a microhabitat within a patch of an individual's home range (4th order selection) to the geographic distribution of the species (0 order selection; Meyer & Thuiller, 2006). Many studies of coyote and red fox habitat selection have examined individual home ranges, focusing on habitat selection at the 2nd or 3rd order scale (e.g., Gosselink et al., 2003; Theberge & Wedeles, 1989). Comparatively less work has examined habitat selection of multiple coyote and red fox populations on a broader spatial scale (1st order selection; e.g. Kays, Gompper, & Ray, 2008; Levi & Wilmers, 2012). Understanding these broad‐scale patterns in canid habitat use is important in landscapes increasingly altered by humans, driving increased human–wildlife interactions and potentially conflict (Gompper, 2002; Weckel, Mack, Nagy, Christie, & Wincorn, 2010).

We examined how broad‐scale landscape characteristics influenced coyote and red fox spatial distribution and relative abundance across rural areas of New York State, USA, using a citizen science dataset collected by volunteer bow hunters. Bow hunters are particularly suited to collect field data for scientific studies; they are camouflaged, observant, and quiet, typically remaining still in one place for several hours at a time, allowing relatively undisturbed observations of wildlife. We predicted that coyote and red fox relative abundance would show positive responses to the amount of human‐dominated land cover (agriculture and low‐density development), with a larger response by red foxes. We also predicted a positive correlation between coyote relative abundance and degree of landscape fragmentation. Finally, we predicted that red fox abundance would be negatively correlated with coyote abundance.

## 2 MATERIALS AND METHODS

### 2.1 Study area

Our study included legally hunted areas across New York State (total area 141,300 km^2^), including state and private land. The vast majority (90%) of hunters in the state hunt on private lands (New York State Department of Environmental Conservation, 2019b). Natural conditions range widely, including rolling hills and plains adjacent to the Great Lakes in the northwest, the Adirondack Mountains in the north, low mountains and lakes in the east, coastal habitat in the southeast, and the Allegheny Mountains and Allegheny plateau in the south and southwest (Nature Conservancy, 2001). Forest land cover types comprise about 56% of state area, agriculture 22%, wetland 8%, high, medium, and low intensity developed areas 10%, and other land‐use types 4% in 2016 (Yang et al., 2018). The state was divided by the New York State Department of Environmental Conservation (DEC) into 92 Wildlife Management Units (WMUs). The average WMU size was 1,364 km^2^ (range 269–7,999 km^2^).

### 2.2 Canid relative abundance data

We obtained hunter counts of coyote and red fox from the New York Bowhunter Sighting Log (hereafter “bowlog”; New York State Department of Environmental Conservation, 2019a). Introduced in 1995, the bowlog is a voluntary daily log kept by bow hunters during the bow hunting season, in which they record the date, location, number of hours hunted, and count of observations of several wildlife species of interest. Individual logs are then compiled by the New York Department of Environmental Conservation to facilitate tracking of long‐term trends in wildlife abundance. We obtained bowlog data from 2004 to 2017 and aggregated counts of coyote and red fox, along with total hours hunted, to the WMU spatial scale for each year. We excluded from the study three WMUs representing the urban areas of New York City and Buffalo, because no hunting was permitted (and thus no bowlog data collected) in these areas. Some records from 2012 were missing, so we excluded the entire year of data. Since the dates of the bow hunting season (and thus the dates of data collection) varied among years and between WMUs, we limited the dataset to logs recorded during 1–25 October each year to reduce potential temporal bias. Hunters did not record the time of day of their observations; we assumed hunter effort was concentrated at the beginning and end of daylight hours to coincide with greater deer activity (e.g., Higdon, Diggins, Cherry, & Ford, 2019), and that this pattern was similar over time and among WMUs. We found that there were some extreme outlier counts in the dataset, likely a result of data input error. To avoid bias introduced by these outliers, we removed from the dataset all counts greater than 10, which represented less than 0.01% of observations in the dataset. We also removed records with missing or unrealistic estimates (>12 hr in a single day) of effort (about 3% of observations).

### 2.3 Covariate data

We obtained land cover rasters at 30‐m resolution for the state of New York for years 2004, 2006, 2008, 2011, 2013, and 2016 from the National Land Cover Database (NLCD; Yang et al., 2018). For each of these years, we calculated the proportion of agricultural land use (classes 81–82), and the proportion of low‐density developed land use (classes 21–22) for each WMU. We did not include medium‐ and high‐density developed areas because they are typically not hunted and did not make up a large percentage of land area. We also excluded areas of open water from the total area in these calculations. We also calculated the perimeter–area ratio of agriculture land‐use patches for each combination of year and WMU as a metric of degree of landscape fragmentation and shape complexity (McGarigal, 2015) using R package landscape metrics (Hesselbarth, 2019). Since not all years in the study (2004–2017) had an available NLCD map, we matched each year to the closest available NLCD year.

### 2.4 Analysis

We fit generalized linear mixed models (GLMMs) to the canid count data in program R (R Core Team, 2019) using R package lme4 (Bates et al., 2019). We fit separate models for coyote and red fox. In each case, the response variable was the number of observations of the species in a given WMU in a given year. Given that the response variables were counts, we examined several possible generalized linear mixed model types suitable for count data, including Poisson, quasi‐Poisson, negative binomial, and Poisson with an observation‐level random effect (OLRE; Bolker, 2020; Elston, Moss, Boulinier, Arrowsmith, & Lambin, 2001; Harrison, 2014; Kéry, 2010). Based on several metrics of model fit, including estimates of marginal and conditional *R* ^2^ obtained using R package MuMIn (Barton, 2019), deviance goodness‐of‐fit tests, and overdispersion tests (Bolker, 2020), we selected a Poisson model with an OLRE (Kéry et al., 2010). Accounting for overdispersion is important in Poisson regression given that ignoring it can result in bias in parameter estimates, incorrect inference, or inflated estimates of *R^2^* (Harrison, 2014). Harrison (2014) reported that use of an OLRE in Poisson regression may not be suitable when overdispersion is due to zero‐inflation. In our dataset, zero‐inflation was likely not an issue; only 3.9% and 11.0% of total observations in a given WMU and year were zeros for coyotes and red foxes, respectively. Thus, we considered the OLRE modeling approach appropriate for our dataset. In both models, we also included log(observation hours) as an offset term in the linear predictor to account for varying effort across space and time, as well as random effects of WMU and year.

Fixed effects in each model included the proportion of WMU area in agricultural land‐use types, the proportion of WMU area in the low‐density developed land‐use types, and the WMU agriculture land‐use area–perimeter ratio. We also included second‐order terms for each of these covariates based on a simple heuristic: If both the first‐ and second‐order terms were statistically significant (*p* < .05), the second‐order term was retained in the model; otherwise it was removed. In the red fox model, we also included a covariate representing coyote relative abundance in the WMU (number of coyotes observed per 100 observation hours). We tested for multicollinearity among these covariates using variance inflation factors (VIF), assuming multicollinearity was not an issue as long as all VIF < 3 (Zuur, Ieno, & Elphick, 2010). To assess model fit, we calculated marginal and conditional *R* ^2^ using R package MuMIn (Barton, 2019). We determined whether estimated fixed‐effect coefficients were statistically different from 0 using Wald tests with *α* = 0.05.

## 3 RESULTS

**FIGURE 1:**
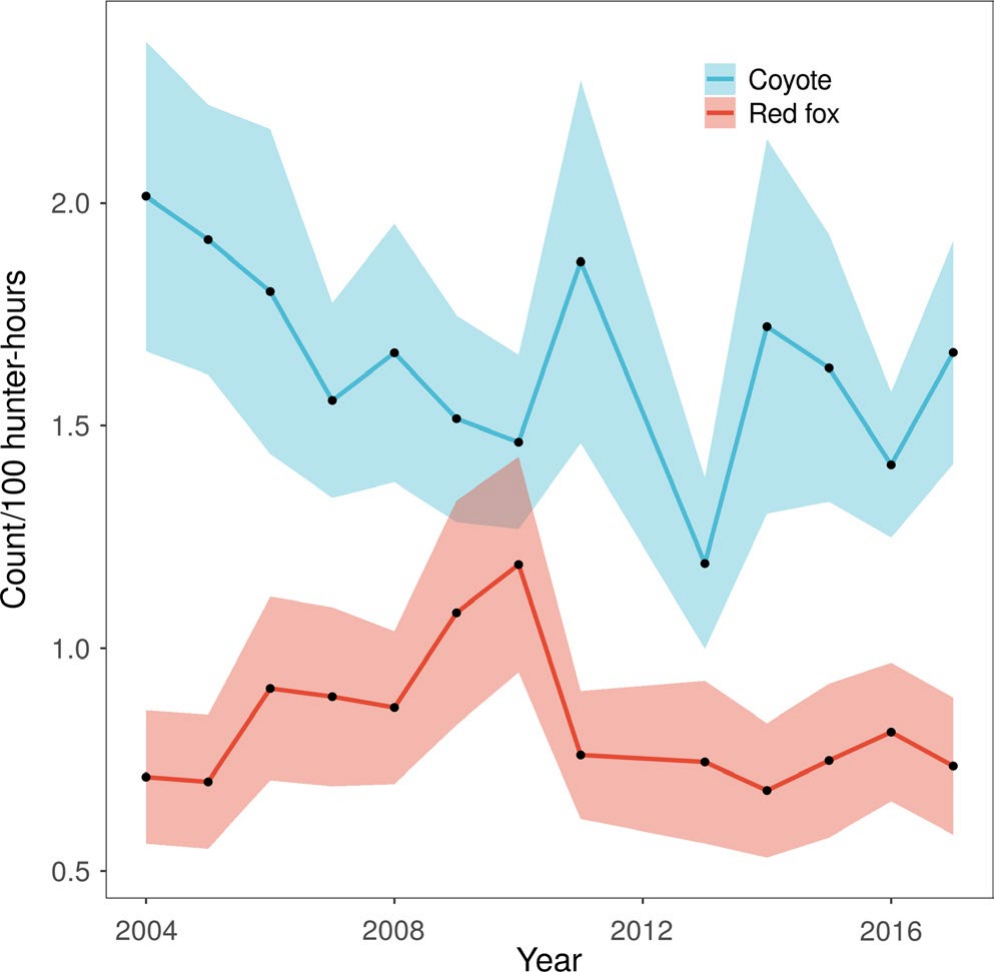
Mean counts of coyote (*Canis latrans*) and red fox (*Vulpes vulpes*) per unit of bow hunter effort in New York State, USA, 2004–2017. Shaded areas represent 95% confidence intervals around the mean

**FIGURE 2:**
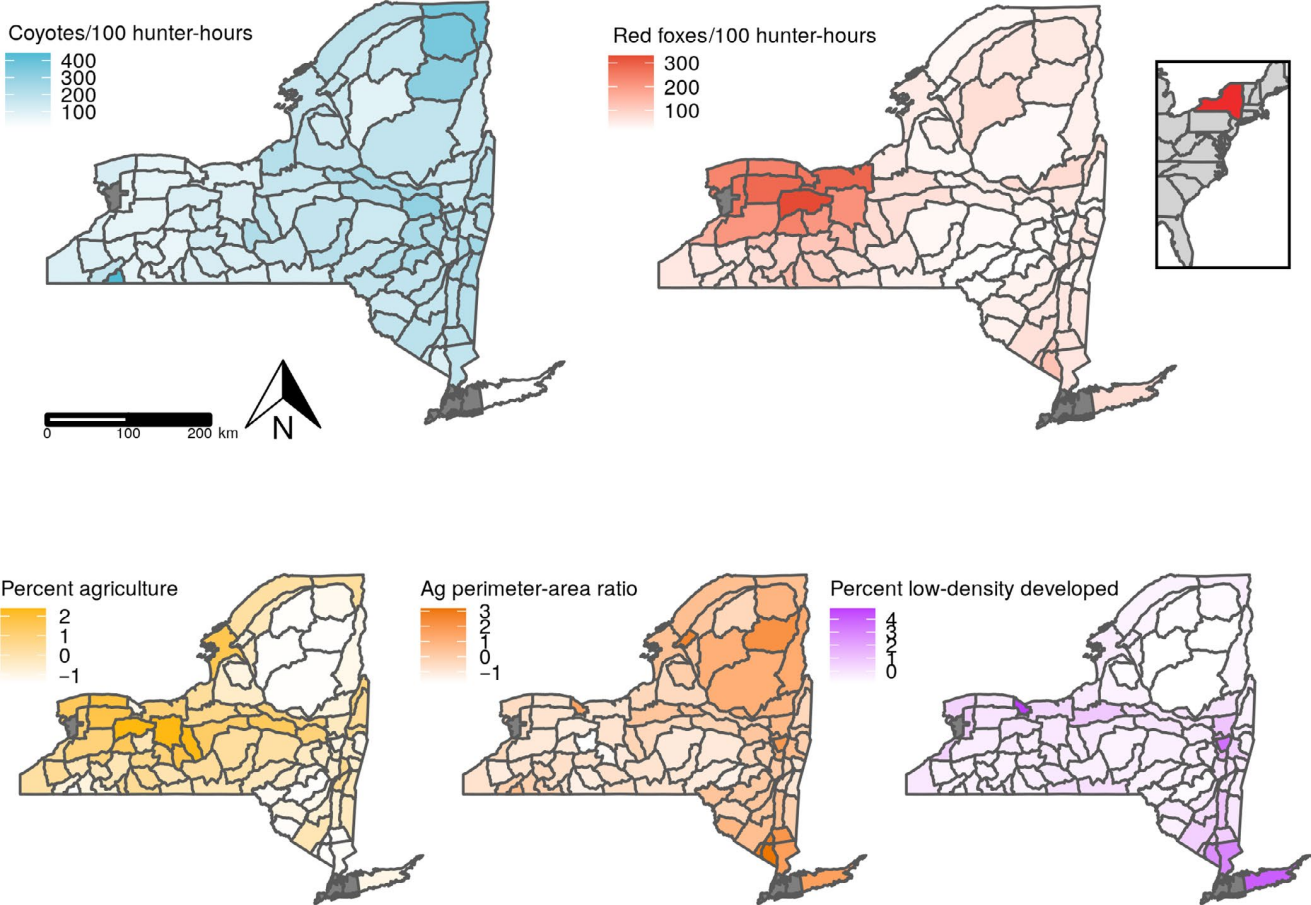
Mean counts of coyote (*Canis latrans*) and red fox (*Vulpes vulpes*) per unit of bow hunter effort (top row) and mean values of land‐use covariates (bottom row) in different regions New York State, USA, 2004–2017

Our final dataset included 342,710 hunter observations (i.e., counts of canids at a specific location on a single day). On average, there were 26,362 observations per year (range: 18,495–34,566). Average observations per WMU (totaled across all years) was 3,851 (range: 377–12,530). Average hunter effort per observation was 3.61 ± 1.86 hr (mean ± *SD*). At least one coyote was counted in 12,047 (3.5%) hunter observations, and at least one red fox was counted in 10,356 (3.0%) hunter observations. Mean total effort per WMU‐year was 1,070 ± 677 hunter‐hours. Across all WMUs, mean count of coyotes per 100 hunter‐hours was 1.62 ± 0.73, and mean count of red foxes per 100 hunter‐hours was 0.82 ± 0.71. While observations varied among years, there were no consistent temporal trends in coyote and red fox counts over time (Figure 1). Coyote and red fox counts, as well as landscape variables, varied regionally across the state (Figure 2).

**FIGURE 3:**
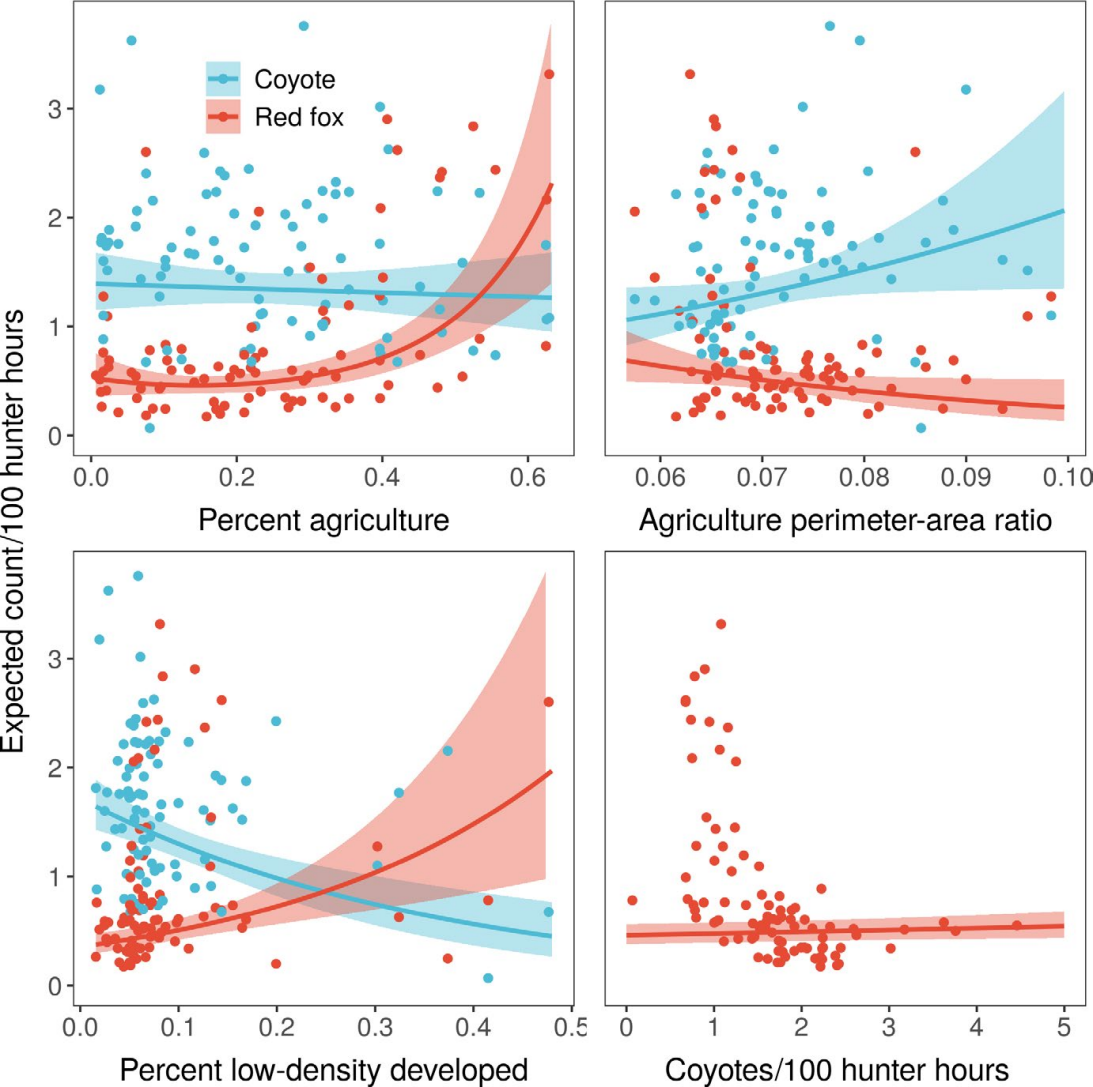
Predicted bow hunter counts per unit of effort of coyote (*Canis latrans*) and red fox (*Vulpes vulpes*) in New York State, USA (2014–2017) across the observed ranges of land‐use covariate values. Shaded areas represent 95% confidence intervals around the predicted value. Points represent mean values for individual wildlife management units based on the raw data. The coyote and red fox predicted count values come from separate generalized linear mixed effects models (see Tables 1 and 2). Note that the predictions do not include variability associated with the random effects in the model

We found a significant negative effect of percent low‐density developed land cover on coyote abundance, with a 1‐standard deviation increase in low‐density developed land cover (a change of about 11%) corresponding to a 21% decline in coyote counts per unit hunter effort (Table 1, Figure 3). Increasing fragmentation of agricultural habitat (i.e, an increase in the perimeter–area ratio of patches in the landscape) had a significant positive effect on coyote counts, with a 1‐*SD* increase in perimeter–area ratio (about 0.008) increasing coyote counts by 14% (Table 1, Figure 3). Total amount of agricultural land cover did not significantly affect coyote counts, and no second‐order terms were retained in the final model based on our heuristic. Marginal *R* ^2^ (variance explained by the fixed effects) for the coyote model was .09, and conditional *R* ^2^ (variance explained by both fixed and random factors) was .87.

**TABLE 1:** Estimated parameters from a generalized linear mixed model of coyote (*Canis latrans*) counts as a function of land‐use covariates in New York State, USA, from 2004 to 2017

Parameter	Estimate	*SE*	*p*
Fixed			
Intercept	0.295	0.053	<.001
Agriculture	−0.025	0.056	.658
Low‐density developed	−0.224	0.054	<.001
Perimeter–area ratio	0.129	0.063	.041
Random			
WMU variance	0.163		
Year variance	0.009		
Observation‐level variance	0.206		

In contrast to coyotes, both low‐density developed and agricultural land cover had positive effects on counts of red fox per unit hunter effort (Table 2, Figure 3). For agricultural cover, both first‐ and second‐order terms were significant and positive, corresponding to an increase in red fox counts with agricultural cover (Figure 3). For low‐density developed cover, only the linear term was included in the model; a 1‐*SD* increase in low‐density developed cover (about 11%) increased red fox counts by 33% (Table 2). Also in contrast to coyotes, perimeter–area ratio of agricultural land cover had a significant negative effect on red fox counts, with a 1‐*SD* increase in the ratio decreasing expected red fox counts by 17% (Table 2, Figure 3). There was no significant relationship between coyote and red fox counts per unit hunter effort (Table 2). Marginal *R* ^2^ for the red fox model was .28, and conditional *R* ^2^ was .87.

**TABLE 2:** Estimated parameters from a generalized linear mixed model of red fox (*Vulpes vulpes*) counts as a function of land‐use covariates in New York State, USA, from 2004 to 2017

Parameter	Estimate	*SE*	*p*
Fixed			
Intercept	−0.719	0.096	<.001
Agriculture	0.208	0.089	.020
Agriculture^2^	0.180	0.060	.003
Low‐density developed	0.287	0.072	<.001
Perimeter‐area ratio	−0.188	0.092	.042
Coyote count	0.047	0.026	.075
*Random*			
WMU variance	0.301		
Year variance	0.022		
Observation‐level variance	0.119		

## 4 DISCUSSION

Using citizen science data, we found differences in the responses of coyotes and red foxes to anthropogenic habitats across New York State. We found mixed support for our prediction that both species would show positive responses to human‐dominated land cover, as development was negatively associated with coyote abundance, but positively associated with red fox abundance. Similar patterns have been documented in other regions where the species are sympatric (Cove et al., 2012; Lombardi, Comer, Scognamillo, & Conway, 2017; Mueller, Drake, & Allen, 2018). Though coyotes can inhabit urban areas, within those areas they typically avoid places of highest human activity (Gehrt, Anchor, & White, 2009; Mueller et al., 2018). Red foxes may use such areas as refuge from coyotes and can reach high densities in urban areas (Gosselink et al., 2003; Mueller et al., 2018).

Coyotes also did not show the predicted response to agricultural habitat. Suitability of agricultural habitat for coyotes varies extensively, which may have contributed to its lack of influence on coyote counts. Coyotes select crops that provide cover and food, such as corn, while avoiding those that do not, such as sorghum and soybeans (Andelt & Andelt, 1981; Gosselink et al., 2003). Row crop fields are dynamic environments in which harvest results in a substantial loss of vegetation over a short time period. This influences the amount of food and cover provided by the crop and can lead to seasonal changes in agricultural habitat use (Andelt & Andelt, 1981). Abrupt losses in vegetation cover can also put coyotes at risk of being killed by humans due to increased visibility and lack of refuge (Van Deelen & Gosselink, 2006). Thus, benefits provided by increased foraging opportunities in agricultural habitats may be offset by increased mortality risk. This fine‐scale selection of agricultural habitat and temporal shifts in its suitability could contribute to lack of coyote response to agricultural habitat across the broad spatial and temporal scales we assessed. These differences in spatial and temporal scales may also be reflected in the overall poor fit (i.e., low marginal *R* ^2^ value) of the coyote model. Coyote count data collected at finer scales (e.g., via camera traps) are likely required to better understand relationships with land‐use variables.

The patterns of coyote abundance we observed may also reflect their population expansion into the state. Coyotes initially colonized New York from the north and circled the Adirondack region before spreading south and west (Fener et al., 2005). This largely corresponds with the densities we documented with highest densities in the north and decreasing density moving south and west (Figure 2). Current coyote abundances across New York may in part be a relic of their historic expansion route, with highest densities in the regions into which they first expanded. Observed lower coyote abundance in low‐density developed areas may also be a function of changes in coyote behavior. Coyotes appear to become more nocturnal in urban and suburban areas (Grinder & Krausman, 2001; Jantz, 2011), which would make them less detectable by bowhunters active between sunrise and sunset, relative to coyotes in rural areas.

In contrast to coyotes, red fox counts were positively associated with both agricultural and low‐density developed land use, supporting our predictions. While it may not be ideal habitat, particularly following harvest (Gosselink et al., 2003), agricultural habitats in New York provide a variety of food for red foxes (Cook & Hamilton, 1944) and open fields are frequently selected for denning because the loose soil facilitates easier digging than in wooded areas (Sheldon, 1950). Our findings match previous work showing red foxes are dependent on human‐dominated habitat types in areas where they overlap with coyotes (Gosselink et al., 2003).

In accordance with our predictions, coyote abundance increased with agricultural perimeter ratio, while red fox abundance declined. Though edge effects are often considered beneficial to carnivores, there is considerable debate regarding this generality. Theberge and Wedeles (1989) also found coyotes to have higher abundances along edges, while others have reported no effect or a negative response to edges (Hinton et al., 2015). Within the same population, resident and transient individuals may have divergent responses to edge habitat (Hinton et al., 2015). Similarly, red foxes have been shown to have higher abundances (Heske, 1995) or no response to edges (Villaseñor, Blanchard, Driscoll, Gibbons, & Lindenmayer, 2015), although we documented a negative response. These disparate responses to habitat fragmentation across their range reiterate the intraspecific habitat selection variation that exists for these species, which has contributed to their widespread geographic ranges. Our results suggest fragmentation by agriculture across New York could augment populations of coyotes but may have the opposite impact on red foxes.

Several mechanisms facilitate coexistence of coyotes and red foxes, which may account for the lack of the predicted inverse relationship between species abundances. Habitat flexibility allows red foxes to shift habitat use to areas avoided by coyotes (Gosselink et al., 2003; Mueller et al., 2018). Red foxes may also alter their diets to avoid competition and potential interactions with coyotes (Theberge & Wedeles, 1989). Additionally, red foxes have smaller home ranges than coyotes, enabling them to persist in relatively small areas between coyote territories (Harrison, Bissonette, & Sherburne, 1989). Coexistence and co‐occurrence at the larger, regional scale (sympatry) may be facilitated by spatial segregation at smaller, local scales (Amarasekare, 2003).

Our study adds to the growing research that employs citizen science data to understand canid ecology. Other studies have used citizen science data to examine the distribution and density of coyotes and red foxes (Mueller, Drake, & Allen, 2019; Scott et al., 2018). Citizen science data have also provided insights into the effects of roads on canids and the factors contributing to human–canid conflicts (Vercayie & Herremans, 2015; Wine, Gagne, & Meentemeyer, 2015). Pack dynamics of a recovering wolf population were studied using scat collected by volunteers (Granroth‐Wildling et al., 2017) and photographs captured by the public provided data on disease spread of urban foxes (Scott et al., 2020). Collectively, this work suggests the potential for substantial advances in understanding human influences on canids using citizen science data. Such potential warrants the investment of resources into refining data collection procedures for citizen science data to glean the maximum insights from this resource.

Due to the characteristics of the surveys used in this study, our data closely match Kamp, Oppel, Heldbjerg, Nyegaard, and Donald (2016) definition of structured citizen science data or monitoring program: All surveys occurred at a specific time of year (October), we accounted for observation effort (hours in the field), and all species observed within a specific list were documented, including common ones (no rarity bias). However, like unstructured data, our data were comprised of opportunistic field observations not randomly located throughout the state; the counts represent relative abundance in areas used by bowhunters and not a random spatial sample within a WMU. Having volunteers document hours spent per survey was key to modeling the sampling process, providing the greatest potential for delivering robust and timely trends based on opportunistic data (Isaac et al., 2014). Population indices derived from chance observations of wildlife per unit of effort are not only of low cost to state wildlife agencies but can provide more accurate assessments than those derived from other types of data, such as spotlighting (Winchcombe & Ostfeld, 2001) or furbearer indices (Obbard et al., 1987), which are influenced by variations in pelt prices (e.g., Conlee & Johnston, 2018). The New York State DEC seeks a more uniform distribution of survey respondents by actively recruiting additional bowhunter volunteers in areas with lower participation (Fies & Norman, 2003).

Voluntary work by hunters and the general public is the foundation of this and many other wildlife monitoring programs around the United States and the globe. Many eastern and midwestern US states monitor target species through hunter observation data that are used for quantifying trends and making management decisions (Fies & Norman, 2003). Outside the United States, Finland has a nationwide hunter‐based monitoring program (the Finish triangle scheme) founded in the 1980s that involves 35 target species; data are used by scientists and the government for multiple management and monitoring goals (Helle, Ikonen, & Kantola, 2016). In addition to hunter‐based programs, many general public‐based programs have remarkably long traditions, such as the Christmas Bird Count in the United States run by the National Audubon Society started in the year 1900, and are the longest‐running and geographically most widespread survey of bird life in the Western Hemisphere (Dunn et al., 2005).

Though our data are informative for understanding canid responses to human development, it is not without limitations. Because hunters are only present during daylight, we do not have data from nighttime hours, which is when these nocturnal species are likely to be most active. This temporal sampling bias could be pertinent because there may be interspecific differences in diel activity patterns of canids in response to human presence. Specifically, people may be more tolerant of red foxes than coyotes, potentially resulting in increased nocturnality by coyotes to avoid humans, but a minimal response by red foxes. If responses differed in this manner, there would be a difference in detectability between species due to red foxes being more active during the day when they would be observed by bow hunters. Additionally, data are limited to the hunting season and do not account for temporal changes that may occur in canid responses to anthropogenic development. We also only have estimates from locations where hunters are active. If canids are using nonhunted areas more intensely, our data may not reflect the true abundances of these species.

Despite its shortcomings, opportunistically gathered data have the potential to make meaningful contributions in biodiversity science and policy‐making (Isaac et al., 2014; Tulloch, Possingham, Joseph, Szabo, & Martin, 2013). Particularly, large citizen science data sets collected at broad spatiotemporal extents can reduce the amount of unsampled variation and lower the risk of false inferences (Bain, Wayne, & Bencini, 2015), and have potential to be integrated with other data sources to model population level dynamics and improve statistical inferences (Sun, Fuller, & Hurst, 2018). Data collected by citizen scientists can be equivalent in reliability and precision to that produced by traditional monitoring approaches (Rafiq et al., 2019). Citizen scientists can provide data used to investigate an array of ecological processes such as the spread of invasive species, wildlife disease dynamics, and the impact of climate change (Dickinson et al., 2012). Participants in citizen science projects often emerge with a greater appreciation of wildlife and nature (Toomey & Domroese, 2013). The engagement of citizens in scientific processes has the potential to combine the collection of useful data with outreach and education, helping to close the knowledge gap between academia and the general public (Jenkins, 2011).

## CONFLICT OF INTEREST

None declared.

## AUTHOR CONTRIBUTION

**Kenneth F. Kellner:** Conceptualization (equal); Data curation (equal); Formal analysis (equal); Investigation (equal); Methodology (equal); Visualization (equal); Writing‐original draft (equal); Writing‐review & editing (equal). **Jacob E. Hill:** Conceptualization (equal); Investigation (equal); Writing‐original draft (equal); Writing‐review & editing (equal). **Mariela G. Gantchoff:** Conceptualization (equal); Investigation (equal); Writing‐original draft (equal); Writing‐review & editing (equal). **David W. Kramer:** Data curation (equal); Methodology (equal); Writing‐review & editing (equal). **Amanda M. Bailey:** Data curation (equal); Methodology (equal); Writing‐review & editing (equal). **Jerrold L. Belant:** Conceptualization (equal); Investigation (equal); Writing‐review & editing (equal).

## Supporting information

Appendix S1

## Data Availability

Data and code used in the paper are available on Data Dryad with https://doi.org/10.5061/dryad.0cfxpnvzs.
