# Supplementary material for: Responses of sympatric canids to human development revealed through citizen science
Source: Ecol Evol. 2020 Jul 15;10(16):8705–14. doi: 10.1002/ece3.6567 (PMC7452815; doi:10.1002/ece3.6567)
Supplement: Supplementary file 1 — Appendix S1 [file ECE3-10-8705-s001.pdf]

Supplementary Material

R Code to fit models from:

Kellner KF, Hill JE, Gantchoff MG, Kramer DW, Bailey AM, Belant JL. (2020). Responses of sympatric canids to human development revealed through citizen science. Ecology and Evolution.

```
#Required libraries
library(tidyverse)
library(lme4)
library(car)
library(MuMIn)
library(psych)

#Overdispersion test function from:
#https://bbolker.github.io/mixedmodels-misc/glmmFAQ.html
overdisp_fun <- function(model) {
  rdf <- df.residual(model)
  rp <- residuals(model,type="pearson")
  Pearson.chisq <- sum(rp^2)
  prat <- Pearson.chisq/rdf
  pval <- pchisq(Pearson.chisq, df=rdf, lower.tail=FALSE)
  c(chisq=Pearson.chisq,ratio=prat,rdf=rdf,p=pval)
}

#Read in raw data (available from Data Dryad)
canid_data <- read_csv("Kellner_canid_data.csv")

#Check correlation among covariates
cor(as.matrix(canid_data[6:8]))
pairs.panels(canid_data[6:8])

#Coyotes------------------------------------------------------------------------

#Fit model
coy_fit <- glmer(coyote ~ ag + urbanL + para_mn + (1|year) + (1|id) +
             + (1|wmu) + offset(log(hours)), data=canid_data,
             family='poisson',na.action='na.fail')
#Save fit
saveRDS(coy_fit, "data/coyote_model.Rds")

#Look at output (Table 1)
summary(coy_fit)

#Check VIF
vif(coy_fit)

#Check fit
r.squaredGLMM(coy_fit)
```

```r
overdisp_fun(coy_fit)
pchisq(deviance(coy_fit), df=df.residual(coy_fit), lower.tail=FALSE)

#Red fox----------------------------------------------------------------------

#Get coyote abundance covariate
coy_sc <- c(scale(canid_data$coyote/canid_data$hours))

#Fit model
rf_fit <- glmer(redfox ~ ag + I(ag^2) + urbanL + para_mn +coy_sc + (1|year) + (1|id)
             + (1|wmu) + offset(log(hours)), data=canid_data,
             family='poisson',na.action='na.fail')
#Save fit
saveRDS(rf_fit, "data/redfox_model.Rds")

#Look at output (Table 2)
summary(rf_fit)

#Check VIF
vif(rf_fit)

#Check fit
r.squaredGLMM(rf_fit)
overdisp_fun(rf_fit)
pchisq(deviance(rf_fit), df=df.residual(rf_fit), lower.tail=FALSE)
```
